# Rate of Growth of Coral

**Published:** 1881-04

**Authors:** 


### RATE OF GROWTH OF CORAL.

Light is thrown on the question of the rapidity with which corals grow, by the case of specimens of living coral which were recently found on the hull of the French man-of-war Dayot, after a cruise of a few months in the South Pacific Ocean. When the vessel reached Tahiti, several corals were discovered growing on the copper sheathing, the longest of which was a *fungia* of discoidal shape nine inches in diameter, and weighing when half dry two pounds and four ounces. The Dayot had entered tropical waters several months before, but had not made a long stay in any harbor until she reached the Gambier Islands, where she remained for two months in the still waters of a coral basin. Thence she sailed direct for Tahiti. A young *fungia* probably became attached to the sheathing of the ship in passing the reef, where the vessel rubbed, and grew to the size and weight it had attained when observed, in nine weeks.—*The Popular Science Monthly.*

